# Gene Expression Profiling in Early Breast Cancer—Patient Stratification Based on Molecular and Tumor Microenvironment Features

**DOI:** 10.3390/biomedicines10020248

**Published:** 2022-01-24

**Authors:** Gyöngyi Munkácsy, Libero Santarpia, Balázs Győrffy

**Affiliations:** 1 TTK Cancer Biomarker Research Group, Institute of Enzymology, Magyar Tudósok Körútja 2, 1117 Budapest, Hungary; gyongyi.munkacsy@med.semmelweis-univ.hu; 2 2nd Department of Pediatrics, Semmelweis University, Tűzoltó Utca 7-9, 1094 Budapest, Hungary; 3 Seagen, Dammstrasse 23, 6300 Zug, Switzerland; liberosantarpia@yahoo.it; 4 Department of Bioinformatics, Semmelweis University, Tűzoltó Utca 7-9, 1094 Budapest, Hungary

**Keywords:** adjuvant chemotherapy, classification, early breast cancer, gene expression profiling, immune-related genes, predictive, prognosis

## Abstract

Patients with early-stage hormone receptor-positive, human epidermal growth factor receptor 2-negative (HER2−) breast cancer (BC) are typically treated with surgery, followed by adjuvant systemic endocrine therapy with or without adjuvant chemotherapy and radiation therapy. Current guidelines regarding the use of adjuvant systemic therapy depend on clinical and pathological factors, such as the morphological assessment of tumor subtype; histological grade; tumor size; lymphovascular invasion; and lymph node status combined with estrogen receptor, progesterone receptor, and HER2 biomarker profiles assessed using immunohistochemistry and in situ hybridization. Additionally, the prognostic and predictive value of tumor-infiltrating lymphocytes and their composition is emerging as a key marker in triple negative (TNBC) and HER2-enriched molecular breast tumor subtypes. However, all these factors do not necessarily reflect the molecular heterogeneity and complexity of breast cancer. In the last two decades, gene expression signatures or profiling (GEP) tests have been developed to predict the risk of disease recurrence and estimate the potential benefit of receiving adjuvant systemic chemotherapy in patients with luminal breast cancer. GEPs have been utilized to help physicians to refine decision-making process, complementing clinicopathological parameters, and can now be used to classify the risk of recurrence and tailoring personalized treatments. Several clinical trials using GEPs validate the increasing value of such assays in different clinical settings, addressing relevant clinical endpoints. Finally, the recent approval of immune checkpoint inhibitors in TNBC and the increasing use of immunotherapy in different molecular BC populations highlight the opportunity to refine current GEPs by including a variety of immune-related genes that may help to improve predicting drug response and finetune prognosis.

## 1. Introduction

Early-stage breast cancer (BC) patients with hormone receptor-positive (HR+) and human epidermal growth factor receptor 2-negative (HER2−) disease are classically treated with surgery, followed by adjuvant endocrine therapies with or without adjuvant chemotherapy (CT) and radiation treatment. The majority of HR+/HER2−/luminal A-like tumors are treated with endocrine therapy alone, or in conjunction with CT in high-risk cases. For HR+/HER2−/luminal B-like tumors, the use of CT is contingent on the expression of hormonal receptors (estrogen/progesterone), proliferative markers (Ki67 expression), tumor burden, genomically-assessed risk, and/or patient preference [1].

In HR+ patients, a prediction algorithm including tumor size, nodal status, tumor grade, proliferative markers, and levels of hormone receptor expression are used to calculate the likelihood of tumor recurrence risk and further support decision making for adjuvant treatments [2]. However, in early-stage BC the benefit of CT does not appear to be strictly related to clinicopathological factors only [3].

Estrogen receptor (ER), progesterone receptor (PR), and HER2 expression are used as molecular markers to stratify patients within specific BC subtypes for treatment and prognosis, but this does not entirely reflect the molecular heterogeneity and complexity of the disease. The additional information provided by multi-gene profiling tests has the capability to advance the accuracy of prognosis and improve risk stratification, identifying patients with a low risk of tumor recurrence, for whom the use of adjuvant CT is not reasonable [4]. Therefore, gene expression profiling (GEP) assays complement prognostic clinicopathological features, improving the accuracy of disease prognosis [5]. In addition, GEP assays tend to correlate with other pathologic features of ER+ tumors like proliferation and grade but providing a more robust reproducible test and independent evidence [6]. Several assays may further classify ER+ BC into different categories defined as high/low or luminal A/B. The biological context, however, is more complex and tumors occur across a full spectrum defined by grade/proliferation and RNA expression profiles [7]. In this review, we evaluate the current evidence of using GEP in clinic and explore and discuss the increasing importance of immune parameters and the possibility to refine current tools with additional immune-GEPs that may help to refine prognosis and provide better predictive tools for early-stage breast cancer.

## 2. Clinical Evidence Supporting the Use of Canonical Gene Expression Signatures 

Various multigene expression signatures have entered main-stream care for luminal early BC patients, providing a prognostic ability beyond that provided by standard clinicopathological features—Oncotype Dx (Genomic Health, Redwood, CA, USA), MammaPrint (Agendia, Amsterdam, The Netherlands), Prosigna (NanoString Technologies, Seattle, WA, USA), Endopredict (Myriad Genetics, Munich, Germany), and Breast Cancer Index (BCI) [8]. For further discussion about the cited molecular tests, technicalities, and performance, we refer to previous comprehensive reviews [5,9]. These molecular tests have been primarily established to classify the risk of disease recurrence and provide information regarding the benefit of receiving adjuvant therapy in early-stage breast cancer. Additional multi-assays based on immunohistochemical and clinicopathological information only but with no molecular components including Mammostrat, eXagenBC, Clinical Treatment Score (CTS5), ProExBr, and 4-marker immunohistochemical score (IHC4) [10,11] will not be a focus of this review.

The clinical utility of MammaPrint, Oncotype Dx, and Prosigna has been explored in the MINDACT [12], TAILOR-x [13] Rx-PONDER [14], and OPTIMA phase III randomized clinical trials [15], respectively. These clinical studies varied in their design, eligibility criteria and endpoints [16]. However, the data from these studies provided high level evidence to support their use in clinic: The MINDACT (Microarray in Node-Negative and 1 to 3 Positive Lymph Node Disease May Avoid Chemotherapy) clinical trial showed excellent 5-year distant metastasis-free (DMFS) survival of 94.7% (95% CI 92.5–96.2) in early-stage BC patients with high-clinical risk and low-genomic risk—assessed by MammaPrint test—who did not receive CT, confirming this patient population could be safely spared from adjuvant CT regardless of their nodal status, hormone-receptor, and HER2 expression. A recent updated analysis confirmed DMFS was 95.1% (95% CI 93.1–96.6) in high-clinical and low-genomic risk patients, and an additional exploratory assessment identified an ‘ultralow’ risk group of patients within the low-risk category with a significant 8-year distant metastasis-free rate of 95–98% [17]. Conversely, the MINDACT study did not prove any substantial difference in outcome in patients treated and untreated with CT in the discordant groups, thus suggesting a limited predictive role for the MammaPrint test signature in this condition [18]. 

The TAILORx (Trial Assigning Individualized Options for Treatment [Rx]) clinical trial demonstrated that early-stage node-negative HR+/HER2− BC patients with Oncotype Dx recurrence score ≤ 25, with a potential concern for intermediate-high risks and premenopausal patients, could be spared from adjuvant chemotherapy. 

The TAILORx trial demonstrated that patients with low-genomic risk (recurrence score ≤ 11) had an excellent prognosis treated with endocrine therapy alone with a 9-year invasive disease-free survival (iDFS) of 84% ± 1.3%. Furthermore, the risk of tumor recurrence and the length of overall survival was significantly associated with genomic recurrence score in the entire population. The TAILORx study showed no significant difference in 9-year iDFS between chemotherapy-treated and untreated patients in the intermediate group (recurrence score 11–25) [19]. Nevertheless, subgroup analyses indicated that patients younger than 50 years of age with a recurrence score between 16–25, and those with a recurrence score between 21–25, appeared to receive a potential benefit from CT as evidenced by a substantial absolute improvement in the distant recurrence-free interval at 9 years of follow-up [19].

The Rx-PONDER (A Clinical Trial RX for Positive Node, Endocrine Responsive Breast Cancer) clinical trial in HR+/HER2− BC patients with one to three positive lymph nodes (nodal stage N1), and an Oncotype Dx recurrence score of ≤25 demonstrated an overall benefit from adding CT to endocrine therapy; however, the study failed to demonstrate a positive and linear interaction between CT benefit and the continuous recurrence score for invasive disease-free survival [14]. The interaction analysis at recurrence score of ≥26 has been shown previously in clinical trials with BC patients either node negative or positive, investigating the additive effects of CT to endocrine therapy. Of note, the study uncovered a significant interaction between CT benefit and menopausal status, where the benefit of CT was exclusively confined to premenopausal women; iDFS at 5 years was 89.0% with endocrine-only therapy and 93.9% with chemoendocrine therapy (hazard ratio, 0.60; 95% CI, 0.43 to 0.83; *p* = 0.002), with a similar increase in distant relapse-free survival (hazard ratio, 0.58; 95% CI, 0.39 to 0.87; *p* = 0.009) [14]. 

An additional prospective phase 3 clinical trial—the WSG-plan B (West German Study Group PlanB)—demonstrated a 5-year iDFS outcome of 94.2% (95%CI = 91.2–97.3%) in clinically high-risk, genomically low-risk, pN0-1 early BC patients treated with adjuvant endocrine therapy only and confirmed that CT could be spared in patients with up to three involved lymph nodes when recurrence score is ≤11. The trial reinforced the use of recurrent score tested with Oncotype Dx with standardized pathology for treatment decisions in HR+/HER2-negative early breast cancer [20]. 

Altogether, the current data support the clinical utility of using genomic predictors in clinical practice, supporting physicians in decision making regarding adjuvant CT in luminal BC patients. The MINDACT clinical trial confirmed the prognostic ability of MammaPrint gene signature for patients with invasive early-stage BC, irrespective of their ER and PR expression, HER2-status, and lymph nodal involvement [12]. Patients with high-risk tumors based on pathological parameters but with low-risk genomic parameters can probably avoid receiving adjuvant chemotherapy. The TAILORx study proposed that adjuvant CT could be safely omitted for HR+/HER2− lymph node-negative patients with a recurrence score ≤ 25, with a possible concern for intermediate-high risks patients (recurrence score 21–25) and for patients aged < 50 years old with a recurrence score of 16–25 [13]. The RxPONDER trial suggested that adjuvant CT can also be omitted in post-menopausal women with one to three positive axillary lymph nodes, and a recurrence score of 25 or lower [14]. 

It is worth mentioning a recent additional study that developed and validated a tool integrating several clinical-pathologic features with the 21-gene expression recurrence score providing an improved patient-specific prognosticator tool for distant recurrence, precisely guiding adjuvant CT in node-negative breast cancer [21].

With the increasing numbers of BC samples analyzed for gene expression, at present, there are multiple freely available online data enabling the validation of new gene expression signatures. Among these the TCGA (https://www.nature.com/articles/nature11412) with more than 1000 patients; the KM-plotter database (https://www.sciencedirect.com/science/article/pii/S2001037021003044) with almost 8000 patients; and the GSE96058 (https://pubmed.ncbi.nlm.nih.gov/32926574/), which includes more than 3000 BC samples. All three datasets (accessed on 5 January 2022) can be evaluated in the KM-plotter website (https://www.kmplot.com, accessed on 5 January 2022), enabling an independent validation of gene expression signatures in different-cohorts of BC patients.

## 3. Use of Gene Expression Signatures Tackling Additional Clinical Endpoints

Additional clinical trials using a variety of GEP tests will assist in addressing additional relevant clinical questions in early BC patients. A full list of additional studies is described in Table 1.

The OPTIMA (Optimal Personalized Treatment of Early Breast Cancer Using Multi-Parameter Analysis) de-escalation, partially blinded, clinical trial will evaluate the ability of the Prosigna PAM50 test to tailor adjuvant therapy for HR+/HER2− high-clinical risk BC patients [22]. Patients aged ≥40 years with resected luminal-type BC and with one to nine involved axillary lymph nodes, or, if node negative, those with a tumor at least 30 mm in diameter will be randomized between standard treatment with CT followed by endocrine therapy or assessed with the Prosigna testing. Patients with tumor having a high-Prosigna Score (>60) will receive standard treatment, whilst those with low-score tumors will receive endocrine therapy only. The study aims to randomize a large number of patients to demonstrate non-inferiority of test directed treatment [22].

The GERICO 11/ASTER 70s is a prospective phase III clinical trial investigating the impact on overall survival of adjuvant CT in elderly women with ER+/HER2− tumors selected with the genomic-grade test prognostic classifier considering competing risks for mortality. Patients classified with high genomic risk are randomized to receive either endocrine therapy alone or CT followed by hormonal therapy; patients with a low genomic-risk are followed in an observational parallel cohort receiving solely hormonal therapy [23].

The EXET (Extended Endocrine Trial) study will evaluate the impact of EndoPredict test to inform treatment decisions for extended endocrine therapy and the subsequent impact on patient outcome. The EndoPredict test predicts late distant recurrence following 5 years of endocrine therapy in ER+/HER2− early-stage patients, with or without adjuvant chemotherapy. The EPclin risk score based on tumor-gene expression, tumor size, and nodal status, assigns patients to high or low risk in relation to metastatic recurrent disease. The primary endpoint is the evaluation of distant recurrence-free survival at 5–10 years in early-stage ER+/HER2− patients with a low-risk score that did not extend endocrine therapy [24].

The RESCUE-Trial will assess DMFS, DFS, and overall survival in patients with HR+/HER2− BC stage I/II and T1 to T3 with zero to three positive lymph nodes and test with EndoPredict score before inclusion. The primary objective is to demonstrate that “low risk” patients treated only with adjuvant endocrine therapy for at least 5 years have a 10-year DMFS of >90%. Secondary endpoints include DMFS, DFS, and overall survival in patients classified as “low risk” vs. “high risk”. In addition, the proportion of patients whose treatment is concordant and non-concordant based on the test results will be analyzed for survival. The prognostic performance of current prognostic factors against survival will also be evaluated [25].

The DxCARTES is an international, open-label, non-comparative, phase II clinical trial that will evaluate the role of the recurrence score (Oncotype Dx) by measuring molecular changes before and after 6 months of receiving palbociclib and letrozole [20]. Principal selection criteria include: (i) Pre- or post-menopausal HR+/HER2− patients with treatment-naïve, Ki67 ≥ 20%, and stage II–IIIB tumors; (ii) pre-treatment recurrence score result ≥ 18; (iii) tissue samples collected at baseline, at C1D14 of treatment, and at time of surgery. Patients will be assigned, based to the pre-treatment recurrent score outcomes, either to Cohort A (recurrence score 18–25) or Cohort B (recurrence score 26–100), and will receive treatment with palbociclib in combination with letrozole, ± LHRH analogs if pre-menopausal, for 24 weeks [26]. Ultimately, breast surgery will be performed within 7 days after six cycles of treatment. The primary objective is to explore the effect of palbociclib in combination with letrozole to induce molecular changes, confirmed by either the post-treatment recurrent score value at surgery, or by pathological complete remission. Secondary objectives include concordance rate among post-treatment recurrent score data and residual cancer disease, Ki67 expression, and preoperative endocrine prognostic index score and overall response rate [26].

The PLATO is a non-randomized, phase II, multi-center prospective clinical trial to increase the rate for breast conserving surgery (BCS) using a personalized neoadjuvant approach in ER+/HER2− women with BC with a measurable tumor size for whom BCS is not feasible. The study will select patients based on histopathologic features of the tumor and MammaPrint testing before receiving neoadjuvant therapy and, therefore, assess the related BCS rate. The size and location of the tumor along with the patient’s breast size will be considered for BCS feasibility. The study will measure the conversion rate for BCS-ineligible patients to BCS-eligible after receiving neoadjuvant chemotherapy. Based on MammaPrint scores, BC patients may receive either CT (high scores) or endocrine therapy (low scores). Premenopausal patients will receive leuprorelin every 4 weeks and letrozole for 16 weeks, whilst postmenopausal women will receive letrozole for 16 weeks. The length of neoadjuvant endocrine therapy can be extended by up to 24 weeks. The primary endpoint is conversion rate in at least 50% of the BCS-ineligible population. The secondary endpoints of the study are actual breast conservation rate, pathologic complete responses, clinical response rate, and disease-free survival [27].

NSABP FB-13 is a phase II, open label clinical trial, which will investigate the molecular and clinical effects on premenopausal patients with ER+/HER2− early invasive BC receiving neoadjuvant endocrine therapy including palbociclib, letrozole, and ovarian suppression. Patients will be assigned into one of two nearly equally sized cohorts based on recurrence score (OnctotypeDx) (recurrence score < 11 and 11 to < 26). Patients in both cohorts will receive palbociclib 125 mg daily for 21 days of a 28-day cycle, letrozole 2.5 mg daily, and goserelin 3.6 mg on D1 of each 28-day cycle. During week 6 of study treatment, patients will undergo two tumor biopsies. Patients from both cohorts with a tumor Ki67 < 10% will continue receiving study treatment for a total of six cycles; those with a persistent Ki67 ≥ 10% will permanently discontinue study treatment and start neoadjuvant CT or receive surgery [28]. 

Samples before treatment and after surgery will be analyzed to identify patients’ subgroups who may receive the highest clinical benefit. Blood samples will be evaluated at baseline, week 4 and 24, for estrone and estradiol levels to determine ovarian suppression. In addition, estradiol levels at week 4 (postmenopausal range) will be collected for the evaluation of additional treatments [28].

The POETIC-A (Peri-Operative Endocrine Therapy for Individualizing Care with Abemaciclib) is a multicentre phase 3 clinical trial enrolling post-menopausal women with operable invasive ER+/HER2− BC, with high 5-year risk of tumor relapse (characterized by high baseline levels of Ki67 ≥ 20%, or tumor size > 5cm, grade 3 tumor, and vascular invasion) after 2 weeks of treatment with aromatase inhibitor (AI) therapy before surgery. Eligible patients are randomized to standard adjuvant endocrine therapy alone or endocrine treatment plus abemaciclib. The aim of the study is to identify patients with an increased risk of developing resistance to endocrine therapy through the measure of Ki67 levels at baseline and after 2 weeks of AI treatment as also investigated in the POETIC trial [29]. Patients in group 1 will receive endocrine therapy only; those in group 2, will receive endocrine therapy combined with abemaciclib. The primary aim is to confirm whether abemaciclib combined with endocrine therapy is more effective than endocrine therapy alone in preventing tumor recurrence [30]. A parallel translational study will prospectively evaluate a predictive gene signature, the Aromatase Inhibitor Resistant-CDK4/6 Inhibitor Sensitive (AIRC-CIS) signature, to determine treatment sensitiveness to abemaciclib [30]. 

## 4. Immune-Related Gene Signatures Complementing Established Molecular Assays

**Figure 1:**
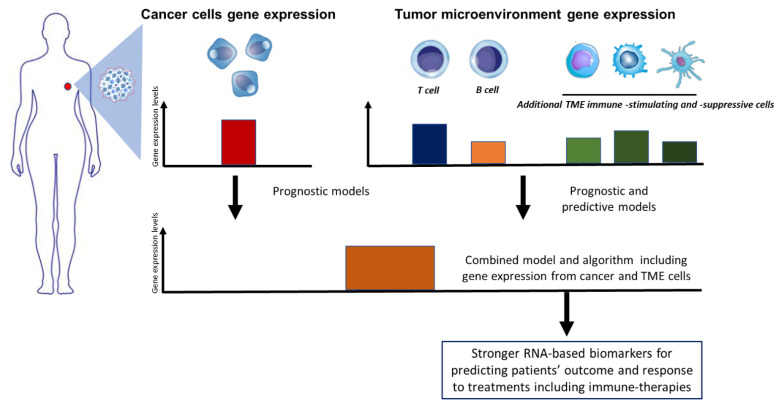
The complex interaction between cancer and the immune tumor microenvironment (TME) influences the outcome of immunotherapy and of several other anti-cancer therapies. A combined model and algorithm that captures both cancer and TME cells (including the immunosuppressive and immunostimulating cells) features through gene expression profiling may result in better prognosticators for measuring outcome and response to therapies.

Current proliferation-related gene signatures mainly associated with replicating tumor cells have shown limitations in predicting prognosis or response to therapies in breast cancer. While assessing tumor infiltrating lymphocytes on tumor slides certainly delivers important prognostic information, a concurrent analysis of both tumor cells and surrounding tumor microenvironment (TME) may help to build up performing gene signatures associated with outcome following therapies [31]. A study schema suggesting a combined gene-based approach capturing cancer cell and TME features is presented in Figure 1.

Some studies have already demonstrated the prognostic or predictive role of gene signatures related to tumor stroma using BC gene expression microarray data. A 26-gene stroma-derived prognostic predictor (SDPP) established using several gene tumor-derived expression datasets was confirmed to be associated with clinical outcome independently of standard clinical prognostic factors and showed an increased accuracy compared to previously published gene predictors. A specific subset of genes related to immune cells and functions, including T cells and natural killer (NK) cell markers representing the T-helper type 1 immune response, were coupled with good prognosis. Conversely, genes associated with hypoxia and angiogenesis, tumor-associated macrophages, as well as a reduction in chemokines expression that stimulates NK cell migration and mediates pro-survival signals in T lymphocytes, were associated with worse clinical outcome [32]. Of note, SDPP predicted clinical outcome in different clinical and molecular BC subtypes, including lymph node negative tumors and showed increased predictive performance particularly in HER2+ tumors. Of interest, the prognostic power of SDPP increased if combined with other established prognostic GEPs [32]. Altogether these data suggest that integrating TME factors into prognostic and prediction models contributes to better understand the relationship between tumor–host interactions and the efficacy of treatment, thus refining and improving the performance of current genomic assays. 

In the same line of research, a different study demonstrated that a 50-gene stromal GEP was able to predict resistance to neoadjuvant CT containing 5-fluorouracil, epirubicin, and cyclophosphamide in ER− BC enrolled within the EORTC 10994/BIG 00-01 clinical trial. The predictive performance of the stromal signature was validated in two cohorts of patients receiving CT but not in untreated patients, confirming the predictive nature of this gene signature [33]. 

In a different study, the investigators developed a stromal-immune index, which was able to classify TNBC patients into two distinct immune phenotypes with different prognosis. Stroma-infiltrating immune cells such as, CD4+ T cells, γδ T cells, and M1-associated macrophages were associated with better outcome, whilst monocytes and M2 macrophages with poor prognosis. The stroma prognosticator was demonstrated to act as an independent prognostic marker and, therefore, serve as a potential predictor for immune activity within the TNBC tumor microenvironment [34]. In line with this study, another research confirmed the importance of the molecular balance and composition of the matrix-producing cells to affect prognosis [35]. 

A comprehensive meta-analysis integrating both clinicopathologic and gene expression data using different molecular BC subtypes demonstrated that proliferation is the underlying feature of most prognostic gene signatures and the most robust biomarker for predicting the clinical outcome in ER+/HER2−, however, the immune response and tumor invasion signals are the key molecular events impacting prognosis in ER−/HER2− and HER2+ molecular subtypes, respectively [36]. 

In line with this study, an integrative analysis demonstrated the importance of immune-related GEP particularly in ER− breast cancer. The analysis showed that, in contrast to ER+ tumors, most of the overexpressed genes in ER−BC were associated with complement activation and immune response signals and better prognosis. Accordingly, the downregulation of an immune response module composed of seven genes was associated with an increased risk for distant metastasis (hazard ratio = 2.02, 95% CI = 1.2–3.4; *p* = 0.009) [37]. Furthermore, the seven-gene immune classifier was confirmed to be a prognosticator in ER− patients independently of lymph node status and presence of lymphocytic infiltration [38]. 

In a different study, using a large group of approximately 600 immune-related genes, the authors defined seven robust metagenes resembling different immune cell types and functions in BC. These metagenes were able to define subgroups of patients with different prognosis. Interestingly, a B cells marker IgG metagene had no significant prognostic value, while a T-cell surrogate marker (lymphocyte-specific kinase metagene) had a strong positive prognostic value in ER− and ER+ tumors overexpressing HER2. Patients with ER− tumors and concurrent high expression levels of IgG and lymphocyte-specific kinase metagenes appeared to have an improved response rate to neoadjuvant chemotherapy [39]. Recently, the role and functions of T-cells in BC-based immunotherapy was extensively reviewed [40].

An additional study identified genes associated with the prediction of distant metastasis in HR-/ER− tumors and compared those with eight others prognostic GEP evaluating potential cancer gene pathway relationships. The study identified a set of 14 prognostic genes including 8 genes linked to immune-inflammatory chemokine regulation and associated with outcome in ER− tumors, including TNBC. The HR−/HER2− gene signature score was shown to outperform other GEPs in the identification of early-stage patients with the higher likelihood to remain free of metastatic disease [41]. Indeed, there was a positive correlation between the ER−/HER2− index and three independent immune-related signatures (STAT1, IFN, and IR), and a negative association between the identified immune signatures and five additional proliferation module-containing signatures (ONCO-RS, MS-14, GGI, CSR/wound, and NKI-70) [41]. The same group developed an optimized five-gene (TNFRSF17, CLIC5, HLA-F, CXCL13, and XCL2) predictor based on interferon-γ and IL-10 signals using previously published GEP data for ER−/HER2−. An integrated cytokine score calculated from the expression of this five-gene panel was shown to act as a predictor of metastatic outcome for patients with early-stage ER−/HER2− tumors independently of lymph node status and adjuvant chemotherapy [42]. 

In TNBC, different TME profiles are linked to different molecular subtypes [43]. High expression of immune metagenes has been associated with an improved prognosis and the expression of inflammation, angiogenesis/hypoxia, and histone-related metagenes with decreased survival in TNBC patients, respectively [44]. Interestingly, high B-cell and low IL-8 metagenes expression has been shown to identify almost one-third of TNBC with a good prognosis, having a relatively low risk of tumor recurrence (hazard ratio = 0.37, 95% CI = 0.22 to 0.61; *p* < 0.001). Of note, the B-cell/IL-8 metagene ratio and lymph nodal status were the only predictors retaining significance after multivariate analysis [44]. In addition, a recent study demonstrated that the presence of T-cell immune response-related genes was associated with DMFS in HR−/HER2+ [45]. Similarly, risk scores driven by the expression of CD2 and MMP11 genes were shown to be independent prognostic factors for DMFS in HR−/HER2+ BC, supporting the prognostic significance of immune genes also in HER2+ patients. Nevertheless, some data have demonstrated that immune gene signatures, such as B cell metagene, may have a potential prognostic significance also in most highly proliferative HR+ tumors, irrespective of ER status, whether most immune gene signatures have been demonstrated to be associated with prognosis in ER−/HER2− tumors. The B-cell metagene, including immunoglobulin genes (IGHG, IGKC, and IGHM), added independent prognostic information to patients having tumors with high proliferative rates (hazard ratio = 0.66; 95% CI = 0.46–0.97) and negative lymph nodes, and it was associated with longer metastasis-free survival regardless of ER/ESR1 and HER2/ERBB2 status [46]. Consequently, altogether these data highlight the importance of the humoral immune system during the metastatic process and prognosis in highly proliferative breast cancers.

A different study reported the presence of 293 overexpressed genes in stroma-enriched core biopsies, including five highly co-expressed metagenes corresponding to different immune functions and extracellular matrix features demonstrating that a B-cell/plasma cell metagene involving the expression of several immunoglobulin genes was associated with prognosis in ER+ tumors with high proliferative rates, but had lower prognostic impact in ER−, and no prognostic value in patients with ER+ tumor with low proliferation activity [47]. These data, if further confirmed, support that the B-cell/plasma cell gene clusters can identify subsets of patients with ER+ and ER− high proliferative tumors with better prognosis [47]. 

The described results are consistent with other studies that used prognostic proliferation- and immunity-related genes to develop a model for lymph node negative BC patients. Proliferation- and immune-related genes showed a negative (*ρ*: −0.603) and positive correlation (*ρ*: 0.243) with survival, respectively, directly reflecting the favorable effect of the immune response against abnormal proliferative activity in ER+ and ER− BC subtypes [48]. The prognostic model showed a reliable and high prognostic performance (hazard ratio = 2.85–3.45) and predicted that nearly 54% of node-negative BC patients (regardless of ER status) will not have tumor recurrence (DMFS) for more than 5 years, with at least 85% of probability to survive [48]. 

More recently, the expression of a prognostic 17 immune-GEP was shown to be associated with DMFS in ER− patients but having high proliferation gene expression only [49]. Subsequent evaluation of blood and BC single-cell RNA-seq datasets revealed that the 17 immunity genes stemmed from inactive tumor-infiltrating lymphocytes that were activated when in contact with tumor cells. The expression of 17 immune-related gene signatures was significantly correlated (*p* < 2.2E-16) with tumor-infiltrating lymphocytes percentage in TNBC patients. A Cox model incorporating BC molecular subtypes, proliferation, and immunity scores (72 gene panel) with significant prediction of outcomes confirmed the impact of tumor immune-related genes on prognosis on different BC gene datasets, suggesting that immune-related GEPs are important parameters to determine prognosis and should be merged into current multi-gene assays to improve risk assessment of distant metastasis [49].

Importantly, recent BC studies have demonstrated the predictive role of immune GEPs or immune-related genes expression in response to drug treatments. A study evaluated tumors of early TNBC patients treated with CT containing NAC (anthracycline, cyclophosphamide, and docetaxel) using a panel of 579 immune-related genes (NanoString nCounter GX Human), and demonstrated that high expressions of T-cell receptor signaling pathway components, Th1-related cytokines, B-cell markers, and cytotoxic molecules were correlated with pathological complete response and longer DFS rate [50]. Conversely, TNBC patients with residual disease showed enhanced expression of neutrophils-related genes [50]. Another recent study using targeted RNA-seq of early BC samples from the neoadjuvant GeparNuevo clinical trial demonstrated that the expression of selected immune-associated signatures was associated with pathologic complete response after CT, however their predictive value related to immune checkpoint inhibition was limited, highlighting the need of further investigations of genes linked to antigen presentation and IFN pathway [51]. The increasing use of RNA-seq assays to profile tumors certainly offers invaluable insights for cancer research and treatments, but at the current stage their use in clinic is partially limited by a few technical aspects described in Table 2. 

Further studies also demonstrated the value of using genomic signatures of immune activation for survival prediction [52] and predicting CT response [53] in TNBC patients.

An additional and different line of research is currently examining the impact of the TME on radiosensitivity in BC patients. Among some studies, a recent research reported the effectiveness of testing the TME (antigen processing and presentation-based immune signature) to predict disease-specific survival and overall response to adjuvant radiotherapy [54]. 

Altogether, the discussed data suggest the importance to consider and further evaluate immune GEPs to refine prognosis and provide better predictive tools for BC patients who may benefit or not from neoadjuvant oradjuvant therapies including CT, different immunotherapies including immune checkpoint inhibitors, or even adjuvant radiotherapy.

## 5. Conclusions

In this review we highlighted the most relevant clinical applications using GEP in the current clinical practice by focusing separately on well-established and widely available molecular tests, and on other runner-up assays with potential clinical relevance. Ongoing clinical trials will potentially unravel further clinical applications of GEPs as well as help to establish the most reliable tests for each patient subpopulation. In the third part of our review, we focus on the emerging and increasing need to refine and implement current molecular tools integrating the TME features, particularly in BC patient cohorts with poorer prognosis like ER−/HER2− and HER2+ patients. Future research using the entire transcriptome might help establishing even more advanced classifications as both coding and non-coding gene variants are associated with outcome in different BC molecular subtypes [55]. Overall, supported by the validated and robust clinical utility and the achieved benefits of these multigenic tests, the landscape of gene expression-based predictive models continues to widen.

## Figures and Tables

**Table 1:** Ongoing clinical studies applying gene expression profiling in early breast cancer.

Clinical Trials (NCT#) and Study Phase	GEP Assay	Breast Cancer Population	Clinical Endpoints	Study Description
OPTIMA (ISRCTN *42400492*) Phase III	Prosigna/PAM50	HR+/HER2−, pN1–2 or pN1mi with pT ≥ 20 mm or pN0 with pT ≥ 30 mm	iDFS	A de-escalation clinical trial to tailor adjuvant therapy for HR+/HER2− high clinical risk breast cancer
RxPONDER (*NCT01272037*) Phase III	Oncotype DX	HR+/HER2−, 1–3 N+, recurrence score ≤ 25	iDFS	Role of recurrent score in predicting benefit of adjuvant chemotherapy in women with LN+ disease
GERICO 11/ASTER 70s (*NCT01564056*) Phase III	Genomic Grade test	HR+/HER2−, N0 or N+, age ≥ 70 years, PS ≤ 2	OS	Adjuvant systemic treatment for ER+/HER2− breast cancers in patients over 70 years of age according to genomic grade index: chemotherapy + endocrine therapy vs. endocrine therapy
EXET (*NCT04016935*) Observational	EndoPredict	HR+/HER2−, stage I–III, 0–3 N+, potential adjuvant chemotherapy, no neoadjuvant chemotherapy	DRFS	Extended Endocrine Trial: A prospective registry study testing the impact of EndoPredict on extended endocrine therapy decisions and patients’ outcomes
RESCUE (*NCT03503799*) Observational	EndoPredict	HR+/HER2−, stage I–III, 0–3 N+	DRFS	Prospective assessment of disease progression in breast cancer patients undergoing EndoPredict test—a care research study
DxCARTES (*NCT03819010*) Phase II	Oncotype DX	HR+/HER2−, Ki67 ≥ 20, stage II-IIIb T2–T4, N0–N2, age ≥ 18, PS ≤ 1	Molecular differences on recurrence score between pre- and post-treatment	Neoadjuvant letrozole + palbociclib in HR+/HER2− patients with stage II–IIIb breast cancer, and pretreatment recurrence scores: 18–25 or 26–100 by Oncotype DX testing. Analysis of recurrent score and pathological feature changes at time of surgery
PLATO (*NCT03900637*) Phase II	MammaPrint	HR+/HER2−, stage I–IIIA, ineligible for BCS, age ≥ 19, PS ≤ 2	Conversion rate	Multicenter study to increase BCS rate with personalized neoadjuvant strategy in HR+/HER2− breast cancer for whom BCS is not feasible. Neoadjuvant chemotherapy and endocrine therapy are conducted on genomic high and low risk patients, respectively
NSABP FB-13 (*NCT03628066*) Phase II	Oncotype DX	HR+/HER2−, T2–T4, candidate for neoadjuvant HT, premenopausal status, PS ≤ 1, recurrence score < 26	Complete cell cycle arrest (percentage of patients with a Ki67 < 2.7%)	Neoadjuvant treatment for premenopausal HR+/HER2− breast cancer patients and evaluation of the clinical and biological effects of palbociclib with ovarian suppression and letrozole
POETIC-A (*NCT04584853*) Phase III	AIR-CIS	HR+/HER2−, postmenopausal status, ≥1.5 cm, grade 3 and/or Ki67 level ≥ 20%	Time to tumor recurrence (local or distant disease)	Preoperative endocrine therapy for individualized therapy with abemaciclib

Aromatase Inhibitor Resistant-CDK4/6 Inhibitor Sensitive (AIR-CIS) test; Breast Conservative Surgery (BCS; DRFS, Distant Recurrence-free Survival; GEP, Gene Expression Profiling; Hormonal Therapy (HT); iDFS, invasive Disease-Free Survival; OS, Overall Survival.

**Table 2:** Comparative approaches for gene expression profile analyses: gene arrays vs. RNA-seq.

	Gene Arrays	RNA-seq
Number of genes	Whole transcriptome	Whole transcriptome
Section of genes	Targeted gene sequences	Entire genes
Bioinformatics complexity	Established tools	Multiple pipelines available
Alternative splicing	Can be included	Feasible but large read counts needed
Strength	Low cost, established pipelines, quality control straightforward	Higher specificity Higher sensitivity for genes expressed either at low or very high level Higher levels of reproducibility
Weakness	Gene sequence must be set upfront cross-hybridization bias	Complex processing, potential GC content bias (mapping ambiguity for paralogous sequences), longer handling time, currently higher cost
Opportunity	High throughput, can be transformed to PCR	Can be used to identify novel transcribed regions, gene variations (e.g., mutations), assess allele-specific expression
Commercially available assays for breast cancer	MammaPrint, Oncotype Dx, Prosigna, etc.	None available yet

## Data Availability

Not applicable.
